# Effects of a Postural Hammock in People with Chronic Neck Pain and Chronic Low Back Pain: A Randomized Controlled Trial

**DOI:** 10.3390/medicina61030502

**Published:** 2025-03-14

**Authors:** José Manuel Delfa-De-La-Morena, Juan-José Mijarra-Murillo, Víctor Navarro-López, Diego Fernández-Vázquez

**Affiliations:** 1 Department of Physiotherapy, Occupational Therapy, Rehabilitation and Physical Medicine, Faculty of Health Sciences, Universidad Rey Juan Carlos, 28922 Madrid, Spain; jose.delfa@urjc.es (J.M.D.-D.-L.-M.); juanjose.mijarra@urjc.es (J.-J.M.-M.); 2 Cognitive Neuroscience, Pain and Rehabilitation in Health Sciences (NECODOR), Faculty of Health Sciences, Universidad Rey Juan Carlos, 28922 Madrid, Spain; 3 Movement Analysis, Biomechanics, Ergonomics, and Motor Control Laboratory (LAMBECOM), Faculty of Health Sciences, Universidad Rey Juan Carlos, 28922 Madrid, Spain

**Keywords:** neck pain, chronic pain, low back pain, musculoskeletal disorders

## Abstract

*Background and Objectives*: Musculoskeletal disorders (MD) affect over 1.7 billion people worldwide, with neck and low back pain being prevalent and debilitating conditions. Current treatments include various interventions, but novel approaches are needed to improve functionality and reduce disability. To evaluate the effects of a postural hammock on pain and functionality in people with chronic neck and low back pain. *Materials and Methods*: A randomized controlled trial was conducted with participants experiencing chronic neck and/or low back pain. They were assigned to either an experimental group using a postural hammock or a control group lying on a mat. Participants underwent five sessions of 10 min each over five consecutive days. *Results*: Forty-three subjects completed the study. While both groups showed improvements, the experimental group exhibited significant increases in hamstring flexibility and pain tolerance, measured through the Visual Analog Scale (VAS) and pressure pain thresholds (PPT). Postural hammock use demonstrated potential benefits in pain management and flexibility compared to conventional methods. *Conclusions*: Using a postural hammock may offer benefits for individuals with chronic back pain. Future research should explore combining hammock therapy with other interventions to enhance outcomes and improve the quality of life for patients with back pain.

## 1. Introduction

Neck pain is defined as pain occurring in the neck with or without irradiation to the upper limbs and lasting at least 1 day [1,2]. According to the World Health Organization (WHO), neck pain was experienced by 222 million people in 2019 [3]. Psychological factors such as depression, anxiety, and stress [4], as well as a sedentary lifestyle, physical inactivity, and the use of computers and cell phones are considered risk factors for neck pain [5].

Nonspecific low back pain (LBP) is defined as pain without a specific cause located between the lower ribs and the gluteal area, with or without irradiation to the lower limbs. LBP was suffered on average by about 619 million people in 2020, and there are estimates that the prevalence in 2050 will be more than 800 million people [6]. Most acute LBP improves rapidly, but in up to 25% of cases it becomes chronic (lasting at least 3 months) [7,8]. Some of the factors associated with chronic LBP are the maintenance of prolonged postures over time, sedentary habits, overweight, and carrying heavy loads. Depression, anxiety, and prolonged stressful situations can also affect the musculoskeletal system and produce chronic LBP [7,9]. LBP is one of the main causes of dysfunction and disability worldwide [10,11], causing numerous hospital admissions [12,13] and important social and health care costs [13,14], being the main cause of work absenteeism and excessive use of therapeutic services [13,15,16].

Neck and low back pain are two of the numerous musculoskeletal disorders (MD) that are characterized by impairments in the muscles, bones, joints, and adjacent connective tissues, leading to temporary or lifelong limitations in functioning and participation. According to WHO, approximately 1710 million people in the world suffer from MD, with the prevalence being higher in high-income countries (36.8%), increasing with age (exceeding 50% between 65 and 69 years of age—52.9%), and in women (22.9% vs. 21.4% men) [1]. MD is the most common cause of disability worldwide, significantly limiting the mobility of the people who suffer from it, being the group of pathologies that cause the most years lived with disability, contributing to 17% of all years lived with disability in the world [2]. The group of MD is heterogeneous, with more than 150 described disorders that affect the locomotor system, such as osteoarthritis, fractures, and nonspecific cervical and lumbar pain. Neck pain ranks fifth in prevalence among MD, and low back pain is considered the most prevalent MD.

The clinical management of back and neck pain includes various approaches, such as medication, education, manual therapy, exercise programs, and psychological interventions, among others [17,18] such as Chinese traditional medicine. A systematic review and meta-analysis carried out by Yuan Q., et al. [19] reported that acupuncture, acupressure, and cupping could improve the pain associated with back and neck pain in the immediate term but not in the long term. A systematic review by Noori et al. [20] showed that there is insufficient evidence that ultrasound therapy is effective in relieving pain in patients with neck or low back pain. A recent Chrocaine overview by Cashin et al. [21] found no strong evidence of any pharmacological intervention reducing the pain intensity of acute or chronic LBP compared to placebo. On the other hand, the exercise programs used in the treatment of LBP (which seem to be beneficial) are varied, including programs such such as strength training, Pilates, and yoga [22,23,24]. A literature review carried out by Paolucci et al. [25] included a total of 26 studies of five different exercise programs (Mckenzie, Feldenkrais, Pilates, Globar Postural Reeducation (GPR) and Propioceptive Neuromuscular Facilitation (PNF), and assessed pain, disability, quality of life, physical function, and phycological aspects for each of them. The authors concluded that any of the five exercise programs could improve quality of life by reducing pain in patients with LBP. However, it was not possible to choose which ones were better than others. Similar conclusions are found in scientific literature. There seem to be no significant differences in the benefits of different types of exercise [26] or in comparison with manual therapy [27,28]. Thus, it is unclear what types or treatments are better for improving the lives of patients with LBP. More studies are needed to implement recommendations of high-quality clinical practice guidelines for the rehabilitation of adults with LBP [18]. Psychological therapies also appear to be of benefit in the treatment of patients with chronic LBP [29], as is stretching the posterior chain musculature [30]. Several studies applying stretching in patients with chronic LBP have shown improvements in pain intensity and flexibility, thus improving functionality [27,31,32]. Castagnoli et al. [33] compared the short- and long-term effects of GPR and individual physical therapy on 103 chonic LBP patients, reporting that both treatments reduced pain in the short term, but that only in the GPR group was pain relief statistically significant in the long term (after one year) in frequency and intensity. However, they did not recommend systematically the long-term use of GPR in patients with chronic LBP due to the higher cost of training physiotherapists to apply it. In this sense, an approach that could be novel to address these problems could be the use of a postural hammock with multiple angles of inclination that promotes relaxation and elongation of the posterior chain musculature, thus being able to simulate the GPR. The hammocks can be adjusted to each subject’s flexibility so that in each session, they can experience a sensation of stretching the posterior chain muscles that is not unpleasant. To our knowledge, we have not found any study in the scientific literature on the use of postural hammocks like the one described in patients with neck or low back pain. Therefore, the aim of this study is to test whether lying in the postural hammock could offer benefits in improving functionality in people with chronic neck and low back pain. Our hypothesis is that the use of hammocks improves flexibility in patients with LBP, producing a better functionality.

## 2. Materials and Methods

### 2.1. Study Design

A randomized controlled trial with two intervention groups was conducted according to the recommendations of the CONSORT guidelines [34]. The protocol was a priori registered in Clinical Trials (ClinicalTrials ID: NCT06226298). Participants were randomly assigned to the control or experimental group by a blinded investigator who was not involved in the intervention. The study was approved by the ethics committee of the Universidad Rey Juan Carlos, with internal reference number 2410202334223, and was carried out in accordance with the Declaration of Helsinki on human research. The participants were informed of the study procedures and agreed to participate in the study by signing an informed consent form. In the present study, the TIDIER (Template for Intervention Description and Replication) checklist was followed to ensure a detailed and consistent description of the intervention performed [35].

### 2.2. Participants

Participants were recruited from the rehabilitation waiting list of the Saluddía Medical Center, located in the Madrid municipality of Rivas-Vaciamadrid. Those on the waiting list were referred to the rehabilitation center by private health insurance companies. The inclusion criteria were: (1) over 18 years of age; (2) to present cervical and/or chronic LBP (pain lasting more than 3 months). The following exclusion criteria were established: (1) having undergone spinal surgery; (2) being diagnosed with a structured vertebral alteration; (3) having had a rupture of the posterior leg musculature in the last year; (4) having some type of injury that prevented the initial evaluation; (5) having practiced vigorous physical exercise in the 24 h prior to the intervention.

### 2.3. Randomization and Allocation Process

After sample selection, a computerized random number generator was used to create simple random assignment sequences. Each participant was assigned to an intervention group regardless of the assignment of previous participants. The randomization process was performed using the Research Randomizer Version 4.0 program. The results will be treated on a modified intention-to-treat basis, in which patients lost to follow-up will be excluded from the final analysis.

### 2.4. Intervention

The research was carried out at the Kybos Prevention S.L. training center, located in Madrid, Spain. Participants in the intervention groups performed 5 active pause sessions of 10 min each for 5 consecutive days. The experimental group lay on the postural hammock, and the control group lay on a mat on the floor. An investigator ensured that the subjects were lying in the hammock or on the mat for 10 min on 5 consecutive days. The people who carried out the intervention were blinded to whether it was a control or experimental group. The research was conducted in open-plan classrooms, with very dim lighting to facilitate relaxation. Both groups were played the same relaxing music during the intervention and were instructed to close their eyes and relax until prompted.

### 2.5. Variables and Measurement Instruments

Prior to the intervention, sociodemographic data were collected from the participants (gender and age). In addition, before the first intervention, participants completed some self-assessment surveys online. The level of physical activity was assessed with the International Physical Activity Questionnaire (IPAQ). The score is calculated through the metabolic equivalent of task (MET) based on the minutes of walking, moderate, and vigorous activity per week, with the total MET subjects classified into a low, moderate, or high level of physical activity [36]. General health status was measured using the “SF-36” questionnaire, with a score from 0–100, where a higher score indicates a more favorable health status [37]. The level of life satisfaction was assessed with the Satisfaction With Life Scale (SWLS), with a score ranging from 5 to 35 points, indicating greater satisfaction with higher scores [38]. Using the Perceived Stress Scale (PSS), the level of perceived stress was assessed. The total score can range from 0 to 56 points, with a higher score indicating a higher level of perceived stress [39]. For the presence of symptoms of anxiety and depression the Hospital Anxiety and Depression Scale (HADS) was used. This scale has two subscales, one for anxiety and one for depression. The score ranges from 0 to 21, with higher scores indicating greater presence of anxiety or depression [40]. The level of disability due to low back and neck pain were measured with the Oswestry [41] scale and the Neck Disability Index [42], respectively; the total score in both scales is reported as a percentage up to 100%, with higher percentages indicating greater disability.

The level of intensity of lumbar and cervical pain was also assessed using the Visual Analog Scale (VAS). On this scale, the subject marks a point on a 10-cm line indicating their pain, resulting in a score from 1 to 10 [43]. The minimally clinically important difference (MCID) of VAS, based on data from adult populations, has been suggested to be a reduction by 1.37 cm [44].

The lumbar and cervical pressure pain threshold (UDP) was assessed with a digital algometer (Model FXD 10, Wagner Instruments, Greewich, CT, USA) that measures pressure in kg/cm^2^. UDP is defined as the minimum pressure necessary for the pressure sensation to begin to be perceived as painful. The UDP of the upper trapezius (right and left) and quadratus lumborum (right and left) muscles was assessed. To improve accuracy, the investigator in charge of taking this measurement marked on the skin the area where the different UDP measurements were taken [42], based on a population of healthy patients, it has been suggested that a PPT change of 17–33% constitutes a relevant change [45].

To evaluate the extensibility of the ischiosural musculature [46], the Sit-and-Reach test (SRT) was performed, evaluating the distance reached on the second attempt. Participants were placed sitting on the floor with extended knees and soles touching the SRT box, raising their shoulders and touching the SRT marker with their fingers. From this position, participants make a slow nonstop trunk flexion until they reach the maximum distance they are capable of. Score changes of between 3–5 cm on this test are estimated to be clinically important in people with low back pain [47].

To evaluate the flexibility of the lumbar spine, the modified Shober test was performed, consisting of beginning from a standing up position, making a trunk flexion trying to touch their feet with their hands. [48]. Some research suggests that the MCID should be approximately 2–3 cm to detect clinically significant changes in lumbar mobility in people with low back pain [49].

The VAS, the UDP, the SRT, and the modified Shober test were performed by two expert evaluators from the research team before the initial intervention, immediately after the first session, after the fifth session, and 72 h after the intervention. The tests were performed in a randomized order and by the same investigator for each test [50].

### 2.6. Sample Size Calculation

The sample size was calculated using the G*Power program version 3.1 for two groups (patients with LBP on a mat and patients with LBP on the postural hammock) with four measurements. The program suggests a total sample of 46 participants (23 in each group) for an analysis of variance using ANOVA with an alpha error of 0.05, a power of 0.9, and an effect size of 0.2. Taking into account a 15% potential loss, it yields a total sample size of 53 participants.

### 2.7. Data Analysis

Data analysis was performed using the SPSS statistical program (version 28.00) [51]. The Shapiro–Wilk test was applied to check the distribution of the variables. The results of the study were represented by descriptive statistics (mean, standard deviation, and range for parametric variables and median or mode, ranges, and quartiles for nonparametric variables).

Analysis of variance (ANOVA) with time (baseline, after 1 and 5 sessions, and 72 h after the intervention) and group (control and intervention) was used to assess between-group differences. The assumption of sphericity was checked with the Mauchly’s test, and, in case of violation (*p* < 0.05), the Greenhouse–Geisser correction was used. Post hoc contrasts were performed to assess intragroup effects at each time point with respect to the baseline [52]. To assess differences between groups in the change at different time points with respect to baseline, an analysis of covariance (ANCOVA) was performed by introducing the baseline NPRS as a covariate in the model [53], because baseline pain may affect the other outcomes. Thus, the effect was “equalized” for the same baseline pain scores. The level of statistical significance was set at *p* < 0.05 for most analyses. The person in charge of data analysis was blinded to the treatment and evaluations performed.

## 3. Results

**Figure 1:**
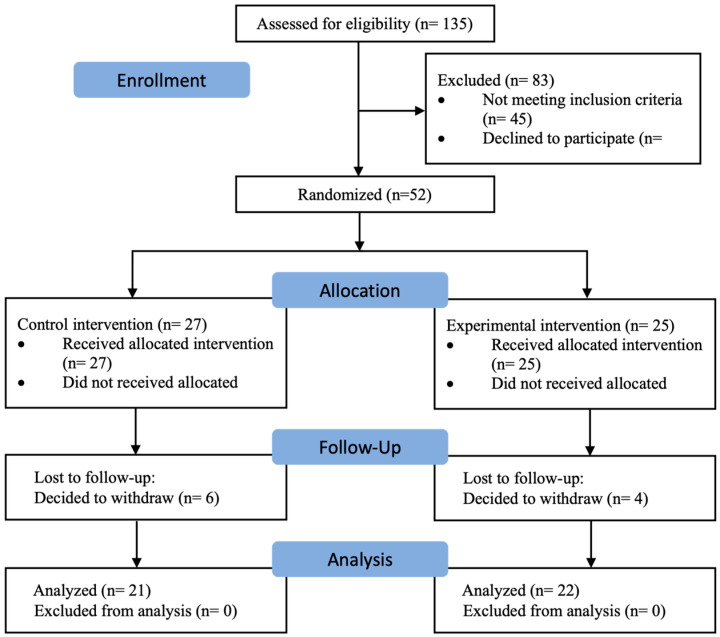
Flow chart.

Forty-three subjects successfully completed the study protocol. Twenty-one were randomly assigned to the control group and 22 to the experimental group (Figure 1). The groups did not differ in the sociodemographic characteristics analyzed, apart from the SWLS with a higher mean score in the control group (Table 1).

### 3.1. Group-Time Interaction Analysis

**Figure 2:**
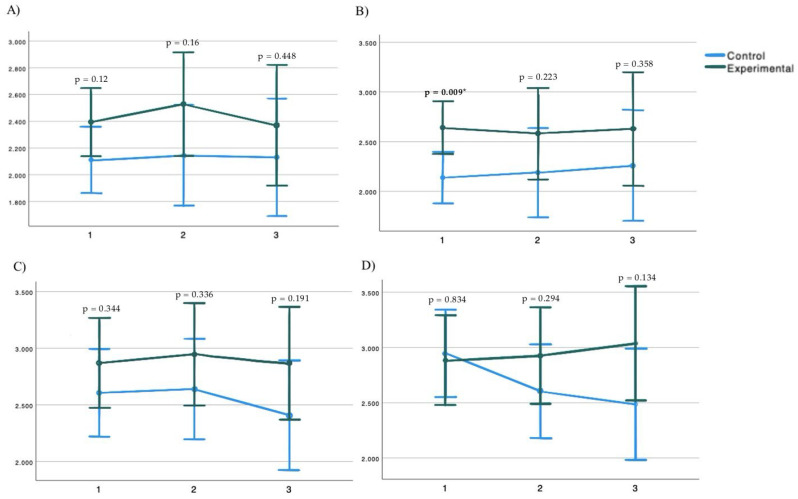
Pain pressure threshold of (**A**) right trapezius; (**B**) left trapezius; (**C**) right quadratus lumborum; (**D**) left quadratus lumborum. * *p*-value was considered significant at <0.05.

**Figure 3:**
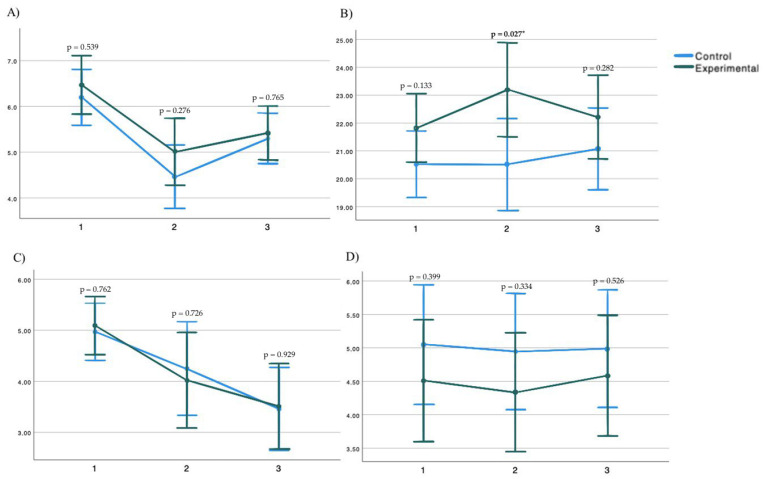
(**A**) Schober test; (**B**) Sit-and-Reach test; (**C**) Cervical Visual Analogue Scale; (**D**) Lumbar Visual Scale. * *p*-value was considered significant at <0.05.

Repeated measurements and ANOVA demonstrated that there was not a significant interaction between group and time in the PPT of the right trapezius muscle (F = 0.694; *p* = 0.526) and left trapezius muscle (F = 1.159; *p* = 0.317), or the PPT of the right quadratus lumborum muscle (F = 0.994; *p* = 0.391) and the left quadratus lumborum muscle (F = 1.636; *p* = 0.196) (Figure 2), the modified Schober test (F = 0.577; *p* = 0.552), the SRT (F = 2.18; *p* = 0.12), the cervical VAS (F = 0.191; *p* = 0.804); and the lumbar VAS (F = 0.78; *p* = 0.488) (Figure 3). Post hoc analysis showed significant differences between the groups in the pain pressure threshold of the left trapezius after 1 session, being higher in the experimental group, and in the SRT being higher after 5 sessions in the experimental group (Table 2).

### 3.2. Within-Subjects’ Analysis

Repeated measurements and ANOVA showed significant effects for the SRT (F = 4.746; *p* = 0.006) and cervical VAS (F = 3.114; *p* = 0.037) in the experimental group, and/or the modified Schober test (F = 3.441; *p* = 0.026) and the cervical VAS (F = 4.17; *p* = 0.012) in the control group. Post hoc analysis demonstrated that in the experimental group, significant differences were obtained in the SRT between the baseline condition and after 1 session, 5 sessions, and the follow-up. Significant differences were also found in the control group, in the cervical VAS between the baseline condition and the follow-up. This difference exceeds the MCID (Table 3).

## 4. Discussion

The present study aimed to evaluate the effects on flexibility and pain intensity of a relaxation protocol of lying in a postural hammock (compared to a control group) in individuals with chronic cervical and LBP. The SRT showed a significant increase in measurements after 5 consecutive days of intervention in the experimental group compared to the control group. Regarding the UPD, a significant increase was observed after the first session in the left trapezius in the experimental group. These results suggest an improvement in the flexibility of the hamstring musculature and a possible improvement in pain tolerance in the experimental group. This improvement was not observed in the control group.

Eighty-five percent of chronic LBP is of nonspecific or unknown cause [54]. Applying the Performance Matrix tests in functional assessments helps to identify weak links of movements and contributes to reducing the weaknesses [55]. Through the elongation of the posterior chain musculature using the postural hammock, benefits may arise in individuals experiencing nonspecific chronic LBP. Using the postural hammock may also introduce appropriate stabilization training, which would undoubtedly contribute to the reduction of the presence of weak links. Sedentary behavior is a risk factor associated with the presence of nonspecific LBP [7,9,56]. Exercise is an important component of LBP management [17,23,32,57]. However, adherence to these programs has been shown to be low to moderate in individuals with cervical or LBP [58]. Hence, it is crucial to explore alternative treatments for these individuals. The utilization of the postural hammock could prove advantageous for sedentary individuals who struggle to comply with exercise-based therapies and lumbar muscle strengthening treatments. Difficulty complying with such therapies and treatments contributed to the 20% (12 participants out of 60) participant loss in the study by Suh et al. [32], in which an improvement in LBP intensity could be seen with a flexibility exercise and with stabilization exercises over six weeks. In our study, there was a lower participant loss of 17.3% (9 participants out of 52), indicating that an intervention such as the hammock may increase adherence to treatment.

The use of a postural hammock could also be relevant for use in occupational settings, as it has been observed that this type of pain is associated with workers who perform heavy lifting, maintain continuous postures (especially in prolonged sedentary jobs), or perform repetitive tasks [59], with an annual prevalence of 40–50% in healthcare workers [60] and 35–50% in office workers [61,62]. The use of the postural hammock for 10 min, 5 days a week, could be easily introduced in companies of different types without influencing the workers’ routine. Workplace interventions have proven to be effective in returning to work, reducing sick leave, and improving work capacity for workers affected by nonspecific LBP, as observed in the meta-analysis by Russo et al. [63]. In this meta-analysis, most interventions consisted of either individual or group exercise programs of a long duration. Despite not specifying the duration of the sessions or the number of weekly sessions, the inclusion of the postural hammock in workplace settings would be easily implementable compared to these types of interventions. Generally, such interventions require more time from companies and greater adherence from workers.

The increase in hamstring muscle flexibility in the experimental group after 5 consecutive days of intervention, compared to the control group, is mainly attributed to the position adopted while lying in the postural hammock. The hammocks were adjusted to each subject’s flexibility so that in each session, they experienced a sensation of stretching the posterior chain muscles that was not unpleasant. The potential improvement in UDP tolerance in the experimental group compared to the control group may be attributed to this increase in flexibility of the posterior chain muscles. Our findings are consistent with a recent trial by Buranruk et al. [64], in which it was observed that stretching has a positive effect on UDP and on improving range of motion. In other research, the performance of muscle stretching exercises has been associated with a reduction in back pain intensity and improvement in functionality, both cervical and lumbar [30,31,64,65,66].

Our results also showed significant intragroup differences in the experimental group, with higher scores in the SRT obtained after lying in the postural hammock for 1 day and increasing over the course of 5 consecutive days of intervention. However, this effect diminished 72 h after the intervention ended, although scores remained higher than at the beginning of the intervention. These results suggest the importance of maintaining long-term adherence to the intervention in order to sustain the flexibility gains achieved and their potential benefits. Lying on a posture saddle seems to enhance flexibility of the hamstring muscles, which seems to increase the pressure pain threshold in patients with chronic back pain.

### 4.1. Implications for Rehabilitation

The postural hammock could be a practical and effective intervention to improve hamstring flexibility and increase pain tolerance in individuals with chronic nonspecific low back pain. This may provide the incorporation of the postural hammock into workplace wellness programs, especially for sedentary workers or those performing repetitive tasks. Its short duration (10 min, 5 days a week) makes it feasible to integrate it into employees’ schedules without significantly altering their routine. Additionally, Parry et al. [67] in their meta-analysis did not report improvements in musculoskeletal symptoms in sedentary employees through interventions such as standing or walking at work. Hence, utilizing the postural hammock could provide an opportunity to explore alternatives and find the best combinations to reduce symptoms and improve functionality in individuals with back pain.

### 4.2. Study Strengths and Limitations

As a strength, this paper tests whether lying in the postural hammock could offer benefits in improving functionality in people with chronic neck and low back pain. This proposal is a novelty in the field and adds information to the existing evidence in the literature. Another strength of this study is that it is a randomized clinical trial (RCT).

### 4.3. On the Other Hand, Several Study Limitations Have Been Identified

There was a greater loss than expected in the calculated sample size, resulting in 43 subjects finally participating in the study out of the 46 calculated, resulting in a lower final size. The short duration of the treatment, with only five sessions, as well as the limited follow-up time, with only one measurement taken 72 h after the fifth session, prevent us from assessing the number of sessions that yield the greatest benefits or the duration of these benefits once the sessions cease.

One potential limitation could be the duration of the sessions, each lasting 10 min, as it might be insufficient to observe significant differences, although it is a more feasible execution time for the intervention to be implemented in companies and thus counteract work absenteeism resulting from back pain. According to Coffeng et al. [68], it is estimated that in Europe, 43% of employees have been absent for at least one day and 23% for up to five days due to pain from musculoskeletal disorders. Therefore, short interventions that can be implemented during working hours may be essential to reducing work absenteeism caused by these disorders. Additionally, the intervention in our study was based on a stretching posture maintained for 10 min. Several studies report muscular benefits (reduction of stiffness, increased extensibility, etc.) with stretching protocols lasting less than 10 min [69,70,71,72]. Finally, no long-term treatment evaluation was performed, which may be of interest for future research.

### 4.4. Future Research

Despite everything, in a future line of research, the most effective session and intervention duration to obtain the greatest benefits should be assessed. Another future line of research would be to assess the effect of implementing combined exercise programs while lying in the postural hammock, taking into account the Performance Matrix test as a previous assessment for design them. [73]. Gobbo et al. [74] showed that a workplace exercise program could reduce symptoms in patients with back pain, and Sowah et al. [75] suggested it could even prevent them. The program could combine strength and flexibility exercises along with body awareness and relaxation techniques, as the benefits of combining different types of exercises and therapies have been reported [76]. In any case, it would be interesting not only to focus on the physical dimension, but also to take into account the psychological and social dimensions that surround a patient with pain [77], which could be another future line of research.

## 5. Conclusions

No significant differences in PPT and VAS were found. However, using the postural hammock for 10 min for 5 consecutive days seemed to improve flexibility measured by SRT on individuals with back pain. Anyway, some limitations of such a small sample are reported. Further studies with greater sample sizes and different time interventions are needed to assess the effects of lying on a postural hammock on patients with back pain.

## Figures and Tables

**Table 1:** Sociodemographic parameters of the participants.

Parameter	Control (*n* = 21)	Experimental (*n* = 22)	*p*
Gender	15 women/6 men	18 women/4 men	
Age	52.73 (10.57)	55.81 (9.25)	0.316
Owestry scale	16.74 (11.51)	20.29 (12.45)	0.357
NDI	24.95 (13.1)	21 (15.25)	0.378
SWLS	25.7 (6.92)	19.76 (9.47)	0.028 *
PSS	26.1 (10.68)	29.71 (6.44)	0.191
HADS Anxiety	8.43 (2.77)	10.2 (3.03)	0.058
HADS Depression	8.76 (2.98)	9.8 (2.35)	0.225
SF36 Physical Functioning	75.79 (18.13)	74.25 (19.49)	0.8
SF36 Physical Role	57.89 (40.01)	48.75 (45.5)	0.51
SF36 Emotional Role	77.19 (38.57)	76.67 (40.61)	0.967
SF36 Energy	50.26 (18.06)	45.75 (16.80)	0.424
SF36 Emotional well-being	64.84 (21.32)	60.6 (16.22)	0.487
SF36 Social Functioning	71.84 (27.05)	73.13 (20.37)	0.868
SF36 Pain	53.03 (24.99)	44.38 (17.01)	0.212
SF36 General Health	51.84 (19.16)	53.42 (18.56)	0.798
IPAQ-SF, MET-min/week	2304.37 (1726.59)	2094.88 (1403.37)	0.675
IPAQ-SF, category			
High	47.4%	23.8%	0.241
Moderate	36.8%	61.9%	0.241
Low	15.8%	14.3%	0.241

HADS: Hospital Anxiety and Depression Scale; Scale IPAQ-SF: International Physical Activity Questionnaire Short Form; MET: metabolic equivalent; min: minute; NDI: Neck Disability Index; SF36: Short Form Health Survey; SWSL: Satisfaction with Life Scale; PSS: Perceived Stress; * *p*-value was considered significant at <0.05.

**Table 2:** Group-time interaction analysis (post hoc analysis).

			Control vs. Experimental		
Parameter	Control (*n* = 21)	Experimental (*n* = 22)	DM	*p*	CI 95%
Right Trapezius PPT 1S	2.110 (0.124)	2.392 (0.127)	−0.281	0.12	−0.639, 0.077
Right Trapezius PPT 5S	2.144 (0.188)	2.529 (0.192)	−0.385	0.16	−0.928, 0.159
Right Trapezius PPT FU	2.13 (0.218)	2.369 (0.223)	−0.24	0.448	−0.871, 0.392
Left Trapezius PPT 1S	2.138 (0.128)	2.641 (0.131)	−0.503	0.009 *	−0.874, −0.132
Left Trapezius PPT 5S	2.187 (0.222)	2.581 (0.227)	−0.394	0.223	−1.039, 0.25
Left Trapezius PPT FU	2.26 (0.277)	2.629 (0.283)	−0.369	0.358	−1.173, 0.434
Right Quadratus Lumborum PPT 1S	2.607 (0.191)	2.87 (0.196)	−0.263	0.344	−0.817, 0.292
Right Quadratus Lumborum PPT 5S	2.639 (0.219)	2.945 (0.224)	−0.306	0.336	−0.941, 0.329
Right Quadratus Lumborum PPT FU	2.408 (0.241)	2.868 (0.247)	−0.46	0.191	−1.158, 0.239
Left Quadratus Lumborum PPT 1S	2.943 (0.197)	2.884 (0.201)	0.059	0.834	−0.511, 0.629
Left Quadratus Lumborum PPT 5S	2.605 (0.211)	2.927 (0.216)	−0.322	0.294	−0.934, 0.29
Left Quadratus Lumborum PPT FU	2.487 (0.251)	3.038 (0.257)	−0.551	0.134	−1.278, 0.176
Modified Schober test 1S	6.199 (0.302)	6.471 (0.316)	−271	0.539	−1.157, 0.614
Modified Schober 5S	4.46 (0.346)	5.014 (0.363)	−0.554	0.276	−1.569, 0.461
Modified Schober FU	5.3 (0.276)	5.42 (0.289)	−0.12	0.765	−0.93, 0.689
SRT 1S	20,513 (0.595)	21.82 (0.609)	−1.307	0.133	−3.03, 0.417
SRT 5S	20,508 (0.819)	23,201 (0.838)	−2.693	0.027 *	−5.064, −0.322
SRT FU	21,074 (0.728)	22,213 (0.745)	−1.138	0.282	−3.246, 0.969
Cervical VAS 1S	4.969 (0.277)	5.088 (2.83)	−0.121	0.762	−0.922, 0.681
Cervical VAS 5S	4.25 (0.455)	4.019 (0.466)	0.23	0.726	−1.087, 1.547
Cervical VAS FU	3.459 (0.402)	3.51 (0.412)	−0.052	0.929	−1.216, 1.113
Lumbar VAS 1S	5.045 (0.442)	4.504 (0.452)	0.541	0.399	−0.742, 1.823
Lumbar VAS 5S	4.942 (0.432)	4.336 (0.442)	0.606	0.334	−0.648, 1.861
Lumbar VAS FU	4.984 (0.437)	4.583 (0.447)	0.401	0.526	−0.866, 1.668

After 1 session; 5S: After 5 sessions; CI: Confidence Interval; DM: Difference of Means; FU: Follow-up; PPT: Pain Pressure Threshold; SRT: Sit-and-Reach Test; VAS: Visual Analogue Scale. * *p*-value was considered significant at <0.05.

**Table 3:** Within-subjects’ analysis (post hoc analysis).

	Pre vs. 1S			Pre vs. 5S			Pre vs. FU			5S vs. FU		
	DM	*p*	CI 95%	DM	*p*	CI 95%	DM	*p*	CI 95%	DM	*p*	CI 95%
Right Trapezius PPT control	0.102	1	−0.247, 0.452	0.067	1	−0.457, 0.592	0.075	1	−0.561, 0.711	0.007	1	−0.536
Right Trapezius PPT Ex	−0.168	1	−0.525, 0.19	−0.304	0.747	−0.841, 0.233	−0.137	1	−0.788, 0.515	0.167	1	−0.341
Left Trapezius PPT control	0.176	1	−0.174, 0.526	0.142	1	−0.474, 0.759	0.065	1	−0.696, 0.827	−0.077	1	−0.556, 0.403
Left Trapezius PPT Ex	−0.337	0.76	−0.695, 0.022	−0.293	1	−0.924, 0.338	−0.336	1	−1.115, 0.443	−0.043	1	−0.534, 0.448
Right Quadratus Lumborum control	0.102	1	−0.425, 0.629	0.096	1	−0.551, 0.742	0.32	1	−0.368, 1.008	0.224	1	−0.402, 0.851
Right Quadratus Lumborum Ex	−0.18	1	−0.719, 0.36	−0.281	1	−0.943, 0.38	−0.197	1	−0.902, 0.507	0.084	1	−0.557, 0.725
Left Quadratus Lumborum control	−0.031	1	−0.581, 0.52	0.341	1	−0.347, 1.030	0.458	0.655	−0.317, 1.233	0.116	1	−0.459, 0.692
Left Quadratus Lumborum Ex	−0.003	1	−0.567, 0.561	−0.081	1	−0.785, 0.624	−0.19	1	−0.983, 0.604	−0.109	1	−0.698, 0.48
Modified Schober test control	−0.468	0.829	−1.327, 0.391	1.391	1	−0.155, 2.937	0.523	1	−0.721, 1.767	−0.868	0.092	−1.819, 0.083
Modified Schober test Ex	−0.8	0.109	−1.701, 0.101	0.525	1	−1.155, 2.146	0.15	1	−1.155, 1.455	−0.375	1	−1.372, 0.622
SRT control	−1.053	0.692	−2.867, 0.761	−1.08	1	−3.561, 1.402	−1.645	0.301	−3.905, 0.614	−0.566	1	−1.903, 0.771
SRT Ex	−2.167	0.014 *	−4.024, −0.31	−3.514	0.003 *	−6.055, −0.974	−2.526	0.025 *	−4.839, −0.213	0.988	0.312	−0.38, 2.356
				**DM**	***p***	**CI 95%**	**DM**	***P***	**CI 95%**			
Cervical VAS control	0.064	1	−0.816, 943	0.834	0.868	−0.721, 2.389	1.631	0.023 *	0.154, 3.108	0.797	0.146	−0.148, 1.742
Cervical VAS Ex	−0.156	1	−1.056, 0.943	0.858	0.858	−0.734, 2.389	1.361	0.1	−0.151, 3.108	0.503	0.941	−0.464, 1.742
				**DM**	***p***	**CI 95%**	**DM**	***p***	**CI 95%**			
Lumbar VAS control	−0.477	1	−1.751, 0.917	−0.403	1	−1.983, 1.176	−0.419	1	−1.916, 1.078	−0.015	1	−1.209, 1.179
Lumbar VAS Ex	0.34	1	−1.025, 1.706	0.603	1	−1.014, 2.220	0.327	1	−1.205, 1.859	−0.276	1	−1.498, 0.946

1S: After 1 session; 5S: After 5 sessions; CI: Confidence Interval; DM: Difference of Means; Ex: Experimental group; FU: Follow-up; PPT: Pain Pressure Threshold; SRT: Sit-and-Reach Test; VAS: Visual Analogue Scale. * *p*-value was considered significant at <0.05.

## Data Availability

All data derived from this research is within the paper.

## Abbreviations

ANOVA: Analysis of Variance; ANCOVA: Analysis of Covariance; CONSORT: Consolidated Standards of Reporting Trials; FU: Follow-up; HADS: Hospital Anxiety and Depression Scale; IPAQ: International Physical Activity Questionnaire; LBP: Low Back Pain; MD: Musculoskeletal Disorders; MET: Metabolic Equivalent; NDI: Neck Disability Index; NPRS: Numeric Pain Rating Scale; PPT: Pain Pressure Threshold; PSS: Perceived Stress Scale; SRT: Sit-and-Reach Test; SWLS: Satisfaction With Life Scale; UDP: Upper Digital Pressure; VAS: Visual Analog Scale; WHO: World Health Organization.
